# Five-mRNA Signature for the Prognosis of Breast Cancer Based on the ceRNA Network

**DOI:** 10.1155/2020/9081852

**Published:** 2020-09-03

**Authors:** Wenjie Shi, Daojun Hu, Sen Lin, Rui Zhuo

**Affiliations:** ^1^Department of Breast Surgery, Guilin TCM Hospital of China, Affiliated to Guang Xi University of Chinese Medicine, Guilin, 541000 Guangxi, China; ^2^Department of Clinical Laboratory, Xinhua Hospital Affiliated to Shanghai Jiao Tong University School of Medicine, Chongming Branch, Shanghai 202150, China

## Abstract

**Background:**

The purpose of this study was to investigate the regulatory mechanisms of ceRNAs in breast cancer (BC) and construct a new five-mRNA prognostic signature.

**Methods:**

The ceRNA network was constructed by different RNAs screened by the edgeR package. The BC prognostic signature was built based on the Cox regression analysis. The log-rank method was used to analyse the survival rate of BC patients with different risk scores. The expression of the 5 genes was verified by the GSE81540 dataset and CPTAC database.

**Results:**

A total of 41 BC-adjacent tissues and 473 BC tissues were included in this study. A total of 2,966 differentially expressed lncRNAs, 5,370 differentially expressed mRNAs, and 359 differentially expressed miRNAs were screened. The ceRNA network was constructed using 13 lncRNAs, 267 mRNAs, and 35 miRNAs. Kaplan-Meier (K-M) methods showed that two lncRNAs (AC037487.1 and MIR22HG) are related to prognosis. Five mRNAs (*VPS28*, *COL17A1*, *HSF1*, *PUF60*, and *SMOC1*) in the ceRNA network were used to establish a prognostic signature. Survival analysis showed that the prognosis of patients in the low-risk group was significantly better than that in the high-risk group (*p* = 0.0022). ROC analysis showed that this signature has a good diagnostic ability (AUC = 0.77). Compared with clinical features, this signature was also an independent prognostic factor (HR: 1.206, 95% CI 1.108−1.311; *p* < 0.001). External verification results showed that the expression of the 5 mRNAs differed between the normal and tumour groups at the chip and protein levels (*p* < 0.001).

**Conclusions:**

These ceRNAs may play a key role in the development of BC, and the new 5-mRNA prognostic signature can improve the prediction of survival for BC patients.

## 1. Introduction

Breast cancer (BC) is a highly invasive and metastatic malignant tumour with a high incidence, which poses a serious threat to women's health and quality of life [1]. In recent years, the incidence and mortality of BC have increased significantly, affecting progressively younger patients [2]. Currently, surgery, chemotherapy, and radiotherapy are the main treatment options for BC [3]. However, due to heterogeneity, breast tumours tend to show high recurrence rates and drug resistance, and the therapeutic effects and prognosis of the disease are not satisfactory [4]. There is an urgent need to develop individualized BC treatment strategies, such as identifying potential biomarkers and therapeutic targets. Therefore, screening for BC biomarkers is important for early diagnosis, improvement of prognosis, and reduction of mortality in BC.

Since Salmena et al. proposed the competitive endogenous RNA (ceRNA) hypothesis, an increasing number of studies have confirmed that ceRNA regulatory networks play an important role in the development of various tumours [5]. Liu et al. demonstrated that the long noncoding RNA (lncRNA) LINC00909 can interact with miR-194 to upregulate the expression of MUCl-C, thereby promoting the proliferation and invasion of glioma cells [6]. Zhao et al. showed that the lncRNA PVT1-214-induced inhibition of miR-128 regulated TrkC and further promoted gastric cancer cell proliferation [7]. Jiang et al. stated that lncRNA TDRG1 regulated the expression of MAPK1 in cervical cancer by targeting miR-326 and promoted the proliferation, migration, and invasion of cervical cancer cells [8]. In addition, studies have shown that pseudogene transcripts also can be used as ceRNAs and participate in tumour regulation. For example, lncRNA PTENP1 can compete with miR-106b to promote PTEN expression and inhibit cell proliferation, thereby inhibiting the development of cervical cancer [9]. The BRAF pseudogene (BRAFP1) can act as an RNA sponge to induce lymphoma in vivo [10]. The regulatory network of ceRNAs involving pseudogenes of the HMGA1 gene plays an important role in the occurrence of breast cancer [11].

In our study, we systematically analysed RNA-seq data on breast cancer (BC) from TCGA database and constructed a BC-related ceRNA network. Finally, a 5-mRNA prognostic signature was constructed based on the mRNAs in the network. The signature-related genes showed stable expression at the chip and protein levels, indicating that this model has value in clinical applications and can be used as a supplement to TNM staging.

## 2. Methods

### 2.1. Data Acquisition and Screening of Differentially Expressed Genes

**Figure 1:**
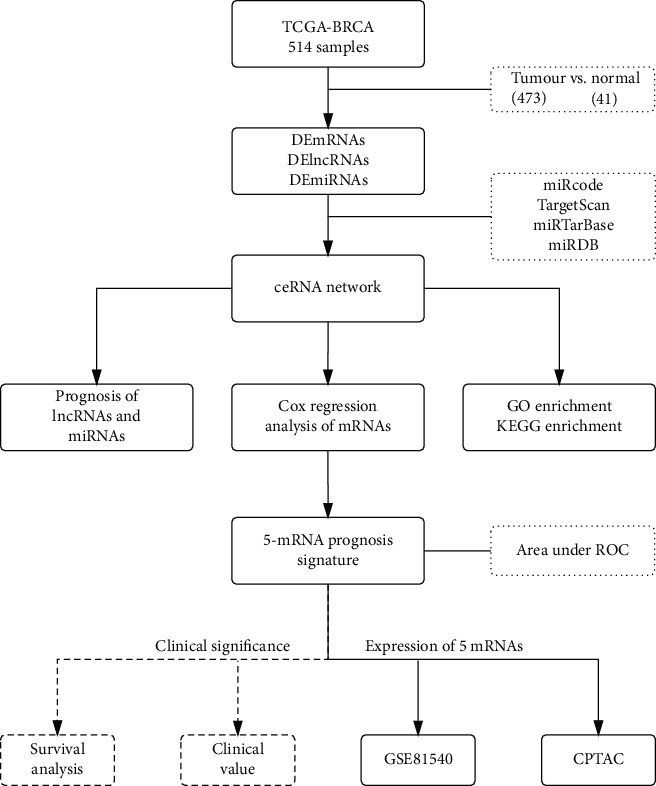
Study design and workflow.

BC data from 514 samples (41 paracancerous tissues and 473 cancer tissues) were downloaded from TCGA website, starting with gene data. The protein-encoded mRNAs and lncRNAs were isolated from the gene data using reannotation, and the differences between mRNAs and lncRNAs were analysed based on *p* < 0.05 and ∣log2 FC | >1. This step was performed using the edgeR package [12]. Similarly, miRNA data from 458 BC samples (8 paracancerous tissues and 450 cancer tissues) were downloaded from TCGA website using the same criteria and methods for differential expression screening. The workflow is shown in Figure 1.

### 2.2. ceRNA Network Construction

To further explore the detailed regulatory mechanism between lncRNAs and miRNAs, we used the miRcode database to compare DElncRNAs and DEmiRNAs, which were selected according to the above criteria. Next, TargetScan, miRTarBase, and miRDB were used to predict the target genes of the miRNAs in the lncRNA-miRNA network. The obtained target genes, DEmiRNAs obtained by the edgeR intersection, and the necessary mRNA components of the lncRNA-miRNA network were determined. Finally, Cytoscape was used to visualize the lncRNA-miRNA regulatory network, which is termed the ceRNA network.

### 2.3. GO Enrichment Analysis and Kyoto Encyclopedia of Genes and Genomes (KEGG) Pathway Analysis

Biological process (BP), cellular components (CC), and molecular function (MF) are usually used as the main domains of Gene Ontology (GO), as they can specifically identify the function of genes in the body. KEGG was used to describe gene participation in signalling pathways to determine the biological functions of different genes in the ceRNA network and their associated signalling pathways. In the present study, the clusterProfiler package was used for GO and KEGG enrichment analyses [13]. The GO terms and KEGG pathway with an adjusted *p* < 0.001 were considered statistically significant.

### 2.4. Identification of Survival-Related RNAs

To accurately explore the mRNAs in the ceRNA network that play an important role in the prognosis of BC patients, we analysed all of the mRNAs by univariate analysis. In univariate analysis, those with *p* < 0.001, which are likely related to prognosis, will be included in the multivariate Cox regression analysis. Furthermore, the mRNA prognostic signature was constructed based on the multivariate regression results. The prognostic value of the lncRNAs and miRNAs in the network was determined using the K-M method.

### 2.5. Validation of Signature

To determine whether the prognosis of patients with different risks, according to mRNA signature stratification, was significant, we used log-rank analysis to compare the high-risk and low-risk groups. Multivariate regression was used to evaluate the value of the risk score compared with other clinical features. In addition, to verify whether the core genes of the signature are significantly expressed at the chip and protein levels, we used the GSE81540 dataset from bc-GeneExMiner V4.4 (http://bcgenex.centregauducheau.fr) and the CPTAC online database (https://proteomics.cancer.gov/data-portal) for validation.

## 3. Results

### 3.1. Screening of Differentially Expressed RNAs

**Figure 2:**
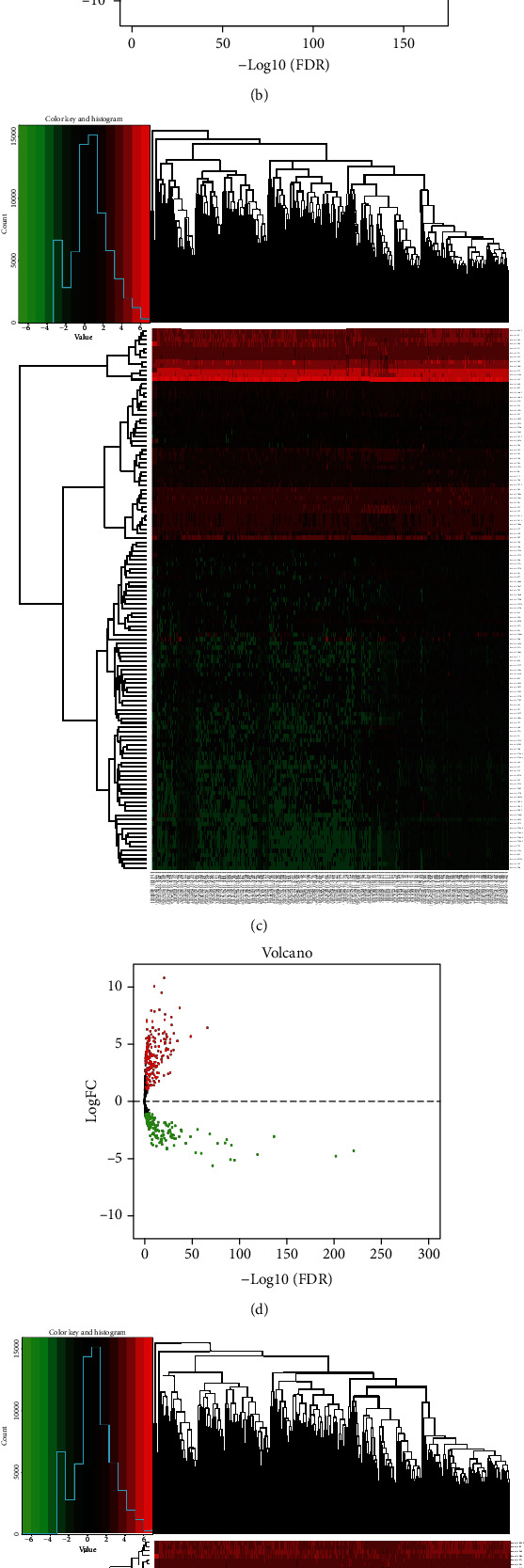
Heat maps of the expression levels of differentially expressed lncRNAs (a), miRNAs (b), and mRNAs (c). The red represents upregulated expression, and the green represents downregulated expression. Volcano plots of the expression levels of differentially expressed lncRNAs (d), miRNAs (e), and mRNAs (f).

A total of 514 breast tissue samples were downloaded from TCGA website for follow-up analysis. Of these, 41 samples were paracancerous tissues and 473 were cancerous tissues. Following standardization with edgeR, the differences between the lncRNAs and mRNAs were analysed based on *p* < 0.05 and ∣log2 FC | >1. A total of 2,966 differentially expressed genes, 2,146 upregulated genes, and 820 downregulated genes were identified (Figures 2(a) and 2(b)). The top 10 differentially expressed lncRNAs are shown in Table 1 (part of the lncRNAs). The same method was used to further process the miRNA data from 458 BC samples downloaded from TCGA database (8 paracancerous tissues and 450 cancerous tissues). A total of 359 differentially expressed miRNAs were screened, of which 210 were upregulated and 149 were downregulated (Figures 2(c) and 2(d)). The top 10 differentially expressed miRNAs are shown in Table 1 (part of the miRNAs). The results revealed 5,370 differentially expressed genes, 3,031 upregulated genes, and 2,339 downregulated genes (Figures 2(e) and 2(f)). The top 10 differentially expressed mRNAs are also shown in Table 1 (part of the mRNAs).

### 3.2. ceRNA Regulatory Networks

The ceRNA network plays an important role in the occurrence and development of breast cancer; thus, elucidation of the internal regulatory relationship of the network is critical. First, 2,966 differentially expressed lncRNAs were compared with 359 differentially expressed miRNAs using 4 databases (miRcode, TargetScan, miRTarBase, and miRDB). The results showed that 13 lncRNAs were compared to 35 miRNAs. The above database was also used to predict the target genes of miRNAs; the predicted results intersected with the differentially expressed mRNAs, and 477 mRNAs were included in the intersection. Next, the database comparison was performed again, and the results showed that 35 miRNAs were compared with 267 mRNAs. Based on the above results, the ceRNA regulatory network was successfully constructed.

### 3.3. mRNA Functional Enrichment Analysis of the ceRNA Networks

**Figure 3:**
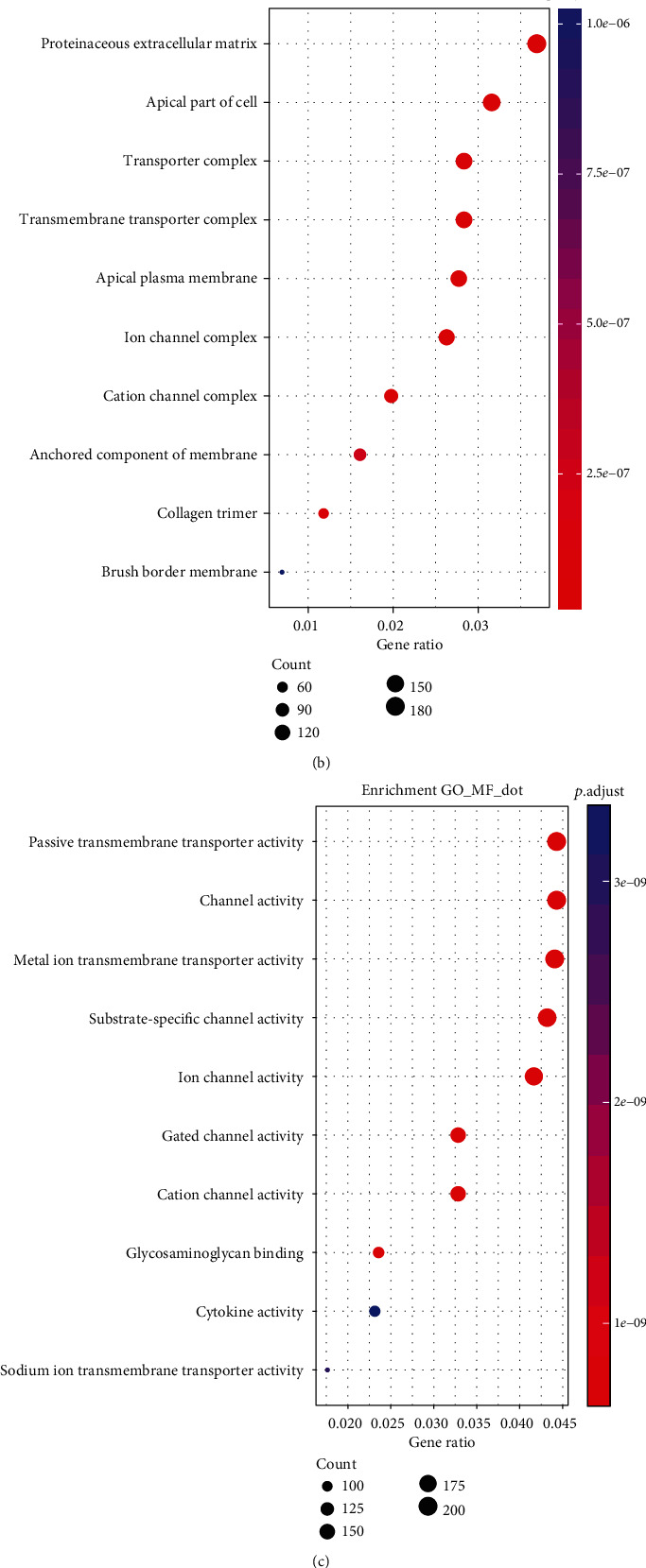
Top 10 pathways identified in the GO and KEGG enrichment analyses in differentially expressed mRNAs. (a) Top 10 pathways of biological process; (b) top 10 pathways of cellular components; (c) top 10 pathways of molecular function; (d) top 10 pathways of KEGG pathways.

Different genes have different important functions in the occurrence and development of diseases, so it is particularly important to understand the function of genes and the signalling pathways in which they may be involved. The clusterProfiler R package was used to analyse the enrichment of differentially expressed genes by GO and KEGG. BP analysis indicated that the genes were enriched in the regulatory system process, circulatory system process, and blood circulation (Figure 3(a)). CC analysis indicated that the genes were enriched in a proteinaceous extracellular matrix, apical cell region, and transporter complex (Figure 3(b)). Regarding MM, these genes mainly participated in passive transmembrane transporter and channel and metal ion transmembrane transporter activities (Figure 3(c)). According to the KEGG pathway analysis, the genes were significantly enriched in neuroactive ligand-receptor interaction, protein digestion and absorption, cytokine-cytokine receptor interaction, and calcium signalling pathway (Figure 3(d)).

### 3.4. Construction of the 5-mRNA Signature and Survival Analysis of ceRNAs

**Figure 4:**
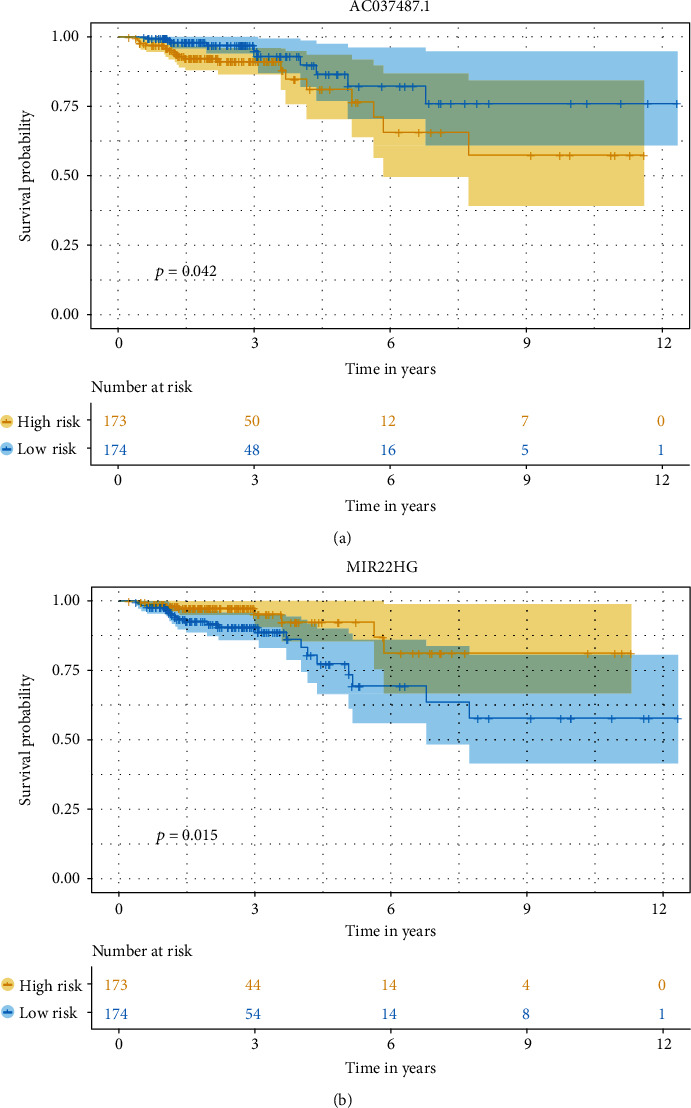
Kaplan-Meier survival curves for 2 lncRNAs AC037487.1 (a) and MIR22HG (b) associated with overall survival in breast cancer.

To accurately identify the potential biological targets of BC and the prognosis-related genes, we combined the mRNAs obtained from the ceRNA networks with prognostic information. In total, 18 genes were found to be significantly associated with overall survival (OS; *p* < 0.05). These genes were subsequently subjected to stepwise multivariate Cox regression analysis. Finally, 5 independent genes were selected, and a gene-based prognostic model was established to estimate the survival of patients using the following equation: risk score = (−0.5814)∗VPS28 + (−0.4078)∗COL17A1 + (0.3795)∗HSF1 + (−0.4378)∗PUF60 + (−0.1876)∗SMOC1 (Table 2). The prognostic effect of lncRNAs and miRNAs on the network cannot be ignored, so we analysed the effects of lncRNAs and miRNAs in the network on prognosis. The results showed that 2 lncRNAs (AC037487.1 and MIR22HG) were associated with OS. High expression of AC037487.1 indicated a poorer prognosis than low expression (Figure 4(a)). In contrast, patients with high expression of MIR22HG often obtained a satisfactory prognosis (Figure 4(b)).

### 3.5. Signature Performance and Verification

**Figure 5:**
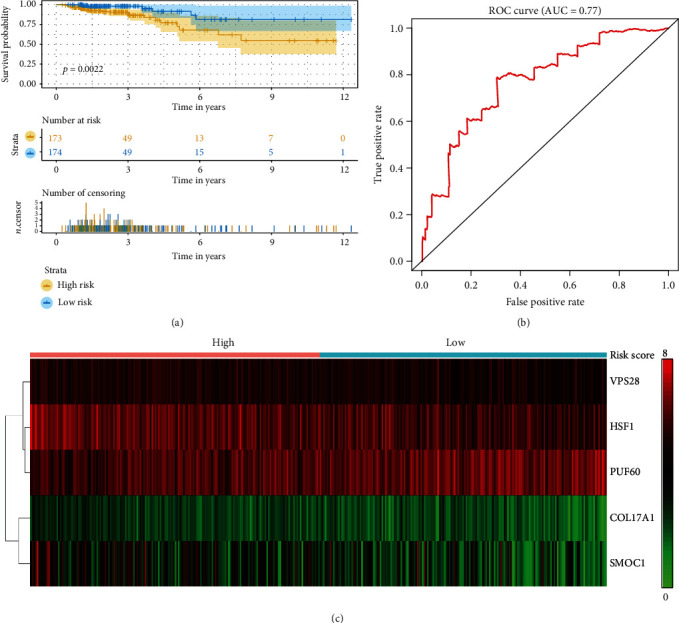
Prognostic evaluation of the 5-mRNA signature in breast cancer patients. (a) Kaplan-Meier analysis of overall survival in breast cancer patients with the 5-mRNA signature. (b) ROC curve analysis of the 5-mRNA signature. (c) The distribution of mRNA-related survival risk scores and heat map of the 5 prognostic mRNAs.

To assess the performance of the model in predicting overall patient survival, we assigned a risk score to each patient using a prognostic model based on the 5 genes. The patients were classified as high risk or low risk based on the median value of the risk score. Kaplan-Meier analysis showed significant differences in the OS curves between the two groups (*p* = 0.0022; Figure 5(a)). ROC curve analysis of the 10-year survival rate was performed to evaluate the predictive potential of the 5 genes. The area under the curve (AUC) for the 5-gene signature-based prognostic model was 0.77 at an OS of 120 months (Figure 5(b)). In addition, the expression levels of these 5 genes from each patient were analysed (Figure 5(c)); green indicates decreased expression and red indicates increased expression. Furthermore, compared with other clinical features, the 5-mRNA signature showed excellent prognostic value and was an independent prognostic factor for BC patients (HR: 1.206, 95% CI 1.108−1.311; *p* < 0.001).

### 3.6. Expression of the 5 mRNAs at the Chip and Protein Levels

**Figure 6:**
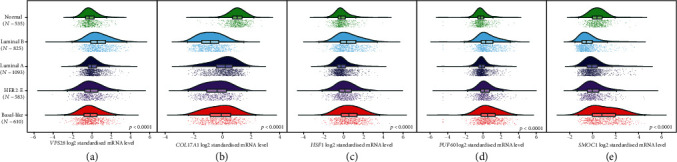
Chip level expression results. Expression of 5 mRNAs (*VPS28*, *COL17A1*, *HSF1*, *PUF60*, and *SMOC1*) in the GSE81450 dataset.

**Figure 7:**
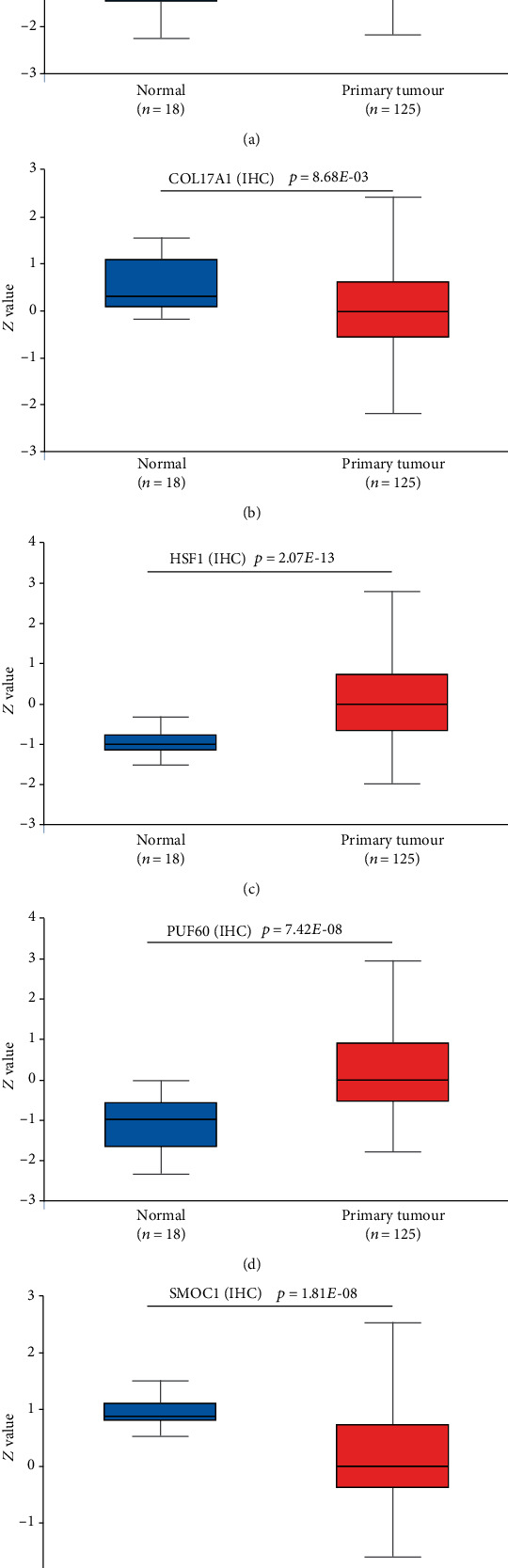
Protein level expression results of 5 mRNAs (*VPS28*, *COL17A1*, *HSF1*, *PUF60*, and *SMOC1*) in the CPTAC database.

To verify whether the 5 genes also have consistent expression results in external databases, we used the GSE81540 dataset and CPTAC to verify expression at the chip and protein levels. The results showed that, regardless of the chip or protein levels, VPS28, HSF1, and PUF60 showed higher expression in tumour tissues than in normal tissues (Figures 6(a), 6(c), and 6(d); Figures 7(a), 7(c), and 7(d)), while COL17A1 and SMOC1 showed significantly higher expression in the adjacent tissues compared with the tumour tissues (Figures 6(b) and 6(e); Figures 7(b) and 7(e)). In addition, according to Figure 6, we found that VPS28 was significantly highly expressed in the luminal B subtype, while the expression pattern of SMOC1 in this subtype was the opposite. However, we also observed that the expression of HSF1 in the luminal A subtype was significantly lower than that of other subtypes, and PUF60 showed a higher expression trend in the TNBC (basal-like) subtype. Notably, compared with that in the TNBC (basal-like) and luminal A subtypes, the expression of COL17A in the HER2+ subtype was significantly reduced. This result implies that these five mRNAs are related to the different BC subtypes. Deregulation of these five mRNAs may become a new breakpoint for the treatment of different BC subtypes.

## 4. Discussion

Analysis of ceRNA networks and gene signatures in tumours is ongoing. However, research has generally focused on one topic, either ceRNAs or a signature. Few studies have investigated both topics. We innovatively built a 5-mRNA signature based on the ceRNA network, and this prediction model is highly accurate and stable.

The mechanism underlying progression and metastasis of BC remains unclear, but with the development of high-throughput sequencing technology, our understanding of this disease is deepening. In a previous study, Lattrich et al. found that systemic miR-195 is a BC-specific biomarker that distinguishes BC from other types of cancer with a sensitivity of 88% and specificity of 91% [14]. Through mouse and human cell experiments, Ma et al. demonstrated that miR-10b is highly expressed in metastatic BC cells and can mediate cell migration in the process of forward transfer and invasion [15]. In addition, Zhang et al. revealed that the miR-191/425 cluster promotes the growth, invasion, and metastasis of breast tumours *in vivo* [16]. These results indicated that miRNAs play an important role in the development of BC. In the present study, a total of 35 miRNAs were included in the ceRNA network, suggesting that these 35 miRNAs may be involved in the development of BC. Unfortunately, when the association between these 35 miRNAs and the OS of patients was evaluated, no miRNAs affecting the prognosis of patients were found in the network. We examined the cause of this result. In Kaplan-Meier analysis, there were too many competitive lncRNAs and downstream mRNAs, which weakened the prognostic value of the miRNAs.

Multiple studies have revealed a close association between lncRNAs and the development of BC [17, 18]. lncRNAs are considered to be potential therapeutic targets for BC and effective predictors of prognosis. Jiang et al. demonstrated that the overexpression of the new lncRNA NLIPMT reduced the phosphorylation of glycogen synthase kinase 3*β*, thereby inhibiting BC metastasis [19]. Shima et al. found that high expression of lncRNA H19 is usually associated with poor prognosis in BC patients, particularly in triple-negative BC [20]. Yu et al. showed that lncRNA HOTAIR regulates the growth, migration, invasion, and apoptosis of BC cells [21]. The ceRNA network explored in the present study contained a total of 13 abnormally expressed lncRNAs. Among them, MIR22HG and ACO37487.1 may be key oncogenes and prognostic markers of BC progression, as they are involved not only in the construction of key nodes in the ceRNA network but also in the OS of BC patients. This conclusion was also confirmed by other researchers. Studies have shown that a high expression of MIR22HG is positively correlated with the OS of patients with hepatocellular carcinoma following hepatectomy, and a high expression of MIR22HG could suppress gastric cancer progression by attenuating NOTCH2 signalling [22, 23]. However, there are no reports of cancer types associated with ACO37487.1, which indicates that ACO37487.1 may be a new target for the diagnosis and treatment of BC.

Due to the differences among individuals, the traditional TNM staging system cannot accurately predict the clinical prognosis of patients [24]. Researchers have attempted to build a new prognostic model to improve the accuracy and sensitivity of survival predictions for BC patients [25, 26]. In the present study, a prognostic risk assessment model for BC was constructed based on Cox multivariate regression results. An AUC of 0.77 indicated that the genetic model has a certain sensitivity and specificity. Five mRNAs in the signature were screened: *VPS28*, *COL17A1*, *HSF1*, *PUF60*, and *SMOC1*. Among them, *COL17A1*, *HSF1*, and *PUF60* are reportedly associated with cancer, as discussed below. COL17A1 encodes the alpha chain of type XVII collagen. COL17A1, as a downstream target of p53, was shown to affect cell migration and invasion, and patients with high expression of this gene often have a better prognosis than those with low expression [27]. Heat shock transcription factor 1 (HSF1), a member of the HSF family of transcription factors, regulates abnormal signals of tumour cells and promotes metastasis and metabolism of BC tumour cells. This finding suggests that HSF1 plays an important role in the occurrence and development of breast tumours [28]. PUF60, an oncogene, is highly expressed in BC tissue samples, and its high expression is closely related to the high lymph node metastasis rate and TNM stage of breast cancer [29]. Nevertheless, the relationships among VPS28, SMOC1, and breast cancer are not currently known, and thus, further investigation in BC patients is required.

There is always an inevitable bias in the data of a single centre. To eliminate this bias and verify the stability of the model, we used an external database to verify the expression of the 5 mRNAs in the signature. The results showed that at both the chip and protein levels, the 5 mRNAs showed stable expression. Thus, these proteins can be detected in BC patients. By applying these results to our model, researchers could obtain a reasonable risk stratification while avoiding excessive medical treatment for low-risk patients and provide a systematic and comprehensive diagnosis and treatment for high-risk patients.

In conclusion, these ceRNAs may play a key role in the development of BC, and the new 5-mRNA prognostic signature was valid and may improve the prediction of survival for BC patients.

## Figures and Tables

**Table 1:** Top 10 differential lncRNAs, miRNAs, and mRNAs for breast cancer.

	Gene name	LogFC	LogCPM	*p* value	FDR
lncRNAs	AC016027.1	-2.721759221	7.807734438	2.09*E*-171	1.57*E*-167
	CDKN2B-AS1	-5.246687203	9.182531501	1.10*E*-140	4.15*E*-137
	LINC01645	-4.614204863	4.578266461	1.56*E*-127	3.91*E*-124
	PP7080	-3.487579802	12.63057754	2.60*E*-122	4.89*E*-119
	AL772337.1	-5.684041773	4.483390005	1.08*E*-117	1.63*E*-114
	AC005358.2	-4.477997898	4.355439858	1.06*E*-114	1.33*E*-111
	AC087379.1	-5.024277942	7.034632957	5.77*E*-114	6.19*E*-111
	AC007182.1	-4.668998329	6.047623463	2.12*E*-107	1.99*E*-104
	AC073283.2	-4.199043651	4.338521941	1.79*E*-106	1.49*E*-103
	HAND2-AS1	-4.60696588	7.977917121	7.87*E*-97	5.91*E*-94
miRNAs	hsa-mir-197	-4.307918408	8.418181254	2.27*E*-224	1.28*E*-221
	hsa-mir-328	-4.785482374	4.592424428	3.29*E*-205	9.25*E*-203
	hsa-let-7d	-3.063424891	9.399730826	1.08*E*-139	2.03*E*-137
	hsa-mir-139	-4.638427903	5.462444197	3.92*E*-122	5.51*E*-120
	hsa-mir-486-2	-5.129991471	5.955407039	9.45*E*-98	1.06*E*-95
	hsa-mir-766	-3.825264088	3.058291436	1.99*E*-94	1.87*E*-92
	hsa-mir-486-1	-5.086542692	5.945615313	2.67*E*-93	2.14*E*-91
	hsa-mir-125a	-3.340248801	8.487687022	2.22*E*-89	1.56*E*-87
	hsa-mir-1306	-3.625960321	3.168653753	1.26*E*-87	7.89*E*-86
	hsa-mir-1976	-3.66459179	3.37270805	1.39*E*-79	7.81*E*-78
mRNAs	CLEC3B	-4.182384289	2.520311404	2.23*E*-247	1.97*E*-243
	CUBN	-5.02609747	1.620310011	3.64*E*-233	2.14*E*-229
	SLC25A34	-4.041302468	1.449619282	2.83*E*-228	1.25*E*-224
	SLC51A	-4.214653548	3.329968713	1.85*E*-221	6.55*E*-218
	CPO	-6.179597798	-0.120368999	1.07*E*-199	3.15*E*-196
	ABCG2	-4.791753484	3.321178055	1.58*E*-191	3.99*E*-188
	TEX11	-4.091470205	-0.14135449	3.68*E*-179	8.13*E*-176
	PHLPP2	-2.588604498	4.751788706	5.80*E*-177	1.14*E*-173
	USP2	-4.047142153	2.525013354	1.61*E*-175	2.85*E*-172
	CA7	-5.664318035	3.497153445	8.00*E*-174	1.28*E*-170

**Table 2:** Five prognostic genes significantly associated with OS with breast cancer patients.

Gene symbol	Coef	Hazard ratio	se (coef)	*z*	*p*
VPS28	-0.5814	0.5591	0.3174	-1.83	0.0670
COL17A1	-0.4078	0.6651	0.2040	-2.00	0.0457
HSF1	0.3795	1.4615	0.2062	1.84	0.0657
PUF60	-0.4378	0.6455	0.1511	-2.90	0.0038
SMOC1	-0.1876	0.8290	0.0715	-2.62	0.0087

## Data Availability

The datasets used and/or analysed during the current study are available from the corresponding author on reasonable request.

## References

[1] Veronesi U., Boyle P., Goldhirsch A., Orecchia R., Viale G. (2005). Breast cancer. *Lancet*.

[2] Anastasiadi Z., Lianos G. D., Ignatiadou E., Harissis H. V., Mitsis M. (2017). Breast cancer in young women: an overview. *Updates in surgery.*.

[3] Shi W., Luo Y., Zhao D., Huang H., Pang W. (2019). Evaluation of the benefit of post-mastectomy radiotherapy in patients with early-stage breast cancer: a propensity score matching study. *Oncology letters.*.

[4] Natarajan K., Xie Y., Baer M. R., Ross D. D. (2012). Role of breast cancer resistance protein (BCRP/ABCG2) in cancer drug resistance. *Biochemical Pharmacology*.

[5] Salmena L., Poliseno L., Tay Y., Kats L., Pandolfi P. P. (2011). A ceRNA hypothesis: the Rosetta stone of a hidden RNA language?. *Cell*.

[6] Liu Z., Lu C., Hu H. (2019). LINC00909 promotes tumor progression in human glioma through regulation of miR-194/MUC1-C axis. *Biomedicine & Pharmacotherapy.*.

[7] Zhao S., Fan N., Chen X., Zhuo C., Xu C., Lin R. (2019). Long noncoding RNA PVT1-214 enhances gastric cancer progression by upregulating TrkC expression in competitively sponging way. *European Review for Medical and Pharmacological Sciences*.

[8] Jiang H., Liang M., Jiang Y. (2019). The lncRNA TDRG1 promotes cell proliferation, migration and invasion by targeting miR-326 to regulate MAPK1 expression in cervical cancer. *Cancer cell international*.

[9] Fan Y., Sheng W., Meng Y., Cao Y., Li R. (2020). lncRNA PTENP1 inhibits cervical cancer progression by suppressing miR-106b. *Artificial Cells, Nanomedicine, and Biotechnology.*.

[10] Karreth F. A., Reschke M., Ruocco A. (2015). The BRAF pseudogene functions as a competitive endogenous RNA and induces lymphoma in vivo. *Cell*.

[11] De Martino M., Forzati F., Marfella M. (2016). HMGA1P7-pseudogene regulates H19 and Igf2 expression by a competitive endogenous RNA mechanism. *Scientific Reports*.

[12] Robinson M. D., McCarthy D. J., Smyth G. K. (2009). edgeR: a bioconductor package for differential expression analysis of digital gene expression data. *Bioinformatics*.

[13] Yu G., Wang L.-G., Han Y., He Q.-Y. (2012). clusterProfiler: an R package for comparing biological themes among gene clusters. *Omics: a journal of integrative biology.*.

[14] Lattrich C., Schüler S., Häring J., Skrzypczak M., Ortmann O., Treeck O. (2014). Effects of a combined treatment with tamoxifen and estrogen receptor *β* agonists on human breast cancer cell lines. *Archives of gynecology and obstetrics.*.

[15] Ma L., Teruya-Feldstein J., Weinberg R. A. (2007). Tumour invasion and metastasis initiated by microRNA-10b in breast cancer. *Nature*.

[16] Zhang X., Wu M., Chong Q.-Y. (2018). Amplification of hsa-miR-191/425 locus promotes breast cancer proliferation and metastasis by targeting DICER1. *Carcinogenesis*.

[17] Liu Y., Sharma S., Watabe K. (2015). Roles of lncRNA in breast cancer. *Frontiers in bioscience(Scholar Edition)*.

[18] Yang Y.-X., Wei L., Zhang Y.-J. (2018). Long non-coding RNA p10247, high expressed in breast cancer (lncRNA-BCHE), is correlated with metastasis. *Clinical & experimental metastasis.*.

[19] Jiang Y., Lin L., Zhong S. (2018). Overexpression of novel lncRNA NLIPMT inhibits metastasis by reducing phosphorylated glycogen synthase kinase 3*β* in breast cancer. *Journal of cellular physiology*.

[20] Shima H., Kida K., Adachi S. (2018). Lnc RNA H19 is associated with poor prognosis in breast cancer patients and promotes cancer stemness. *Breast cancer research and treatment.*.

[21] Yu Y., Lv F., Liang D. (2017). HOTAIR may regulate proliferation, apoptosis, migration and invasion of MCF-7 cells through regulating the P53/Akt/JNK signaling pathway. *Biomedicine & Pharmacotherapy.*.

[22] Dong Y., Yan W., Zhang S.-L. (2017). Prognostic values of long non-coding RNA MIR22HG for patients with hepatocellular carcinoma after hepatectomy. *Oncotarget*.

[23] Li H., Wang Y. (2019). Long noncoding RNA (lncRNA) MIR22HG suppresses gastric cancer progression through attenuating NOTCH2 signaling. *Medical Science Monitor*.

[24] Cuccurullo V., Mansi L. (2011). *AJCC Cancer Staging Handbook: From the AJCC Cancer Staging Manual*.

[25] Balachandran V. P., Gonen M., Smith J. J., DeMatteo R. P. (2015). Nomograms in oncology: more than meets the eye. *The lancet oncology*.

[26] Brentnall A. R., Cuzick J. (2020). Risk models for breast cancer and their validation. *Statistical science: a review journal of the Institute of Mathematical Statistics.*.

[27] Yodsurang V., Tanikawa C., Miyamoto T., Lo P. H. Y., Hirata M., Matsuda K. (2017). Identification of a novel p53 target, COL17A1, that inhibits breast cancer cell migration and invasion. *Oncotarget*.

[28] Vydra N., Janus P., Toma-Jonik A. (2019). 17*β*-estradiol activates HSF1 via MAPK signaling in ER*α*-positive breast cancer cells. *Cancers*.

[29] Sun D., Lei W., Hou X., Li H., Ni W. (2019). PUF60 accelerates the progression of breast cancer through downregulation of PTen expression. *Cancer management and research.*.

